# Knowledge and Education on Physical Activity Health Benefits and Prescription Principles Among Greek Medical Students

**DOI:** 10.3390/bs15070925

**Published:** 2025-07-09

**Authors:** Eirini Kyriakoulakou, Apostolos Z. Skouras, Charilaos Tsolakis, Panagiotis Koulouvaris, Anastassios Philippou

**Affiliations:** 1 1st Department of Orthopaedic Surgery, School of Medicine, National and Kapodistrian University of Athens, 12462 Athens, Greece; e.kyriakoulakou@gmail.com (E.K.); apostolis.sk@gmail.com (A.Z.S.); tsolakis@phed.uoa.gr (C.T.); info@drkoulouvaris.gr (P.K.); 2 Sports Performance Laboratory, School of Physical Education & Sports Science, National and Kapodistrian University of Athens, 17237 Athens, Greece; 3 Department of Physiology, Medical School, National and Kapodistrian University of Athens, 11527 Athens, Greece

**Keywords:** lifestyle medicine, medical curriculum, medical education, physical education, WHO guidelines

## Abstract

Physical activity (PA) is widely recognized as a therapeutic intervention for numerous non-communicable diseases. This study assessed Greek medical students’ knowledge and education on PA across seven medical schools. A structured questionnaire was distributed electronically to all medical schools across Greece, with 135 students responding (67.4% female). Among respondents, 78.5% reported being taught about PA benefits, and 77.8% felt prepared to discuss them with patients. However, 30.2% had received less than two hours of formal PA education. Only 25.2% were aware of the World Health Organization (WHO) and Greek Central Board of Health (KESY) recommendations, while 81.5% expressed the need for additional education on PA and its role in health. Students who were taught about PA were more likely to address exercise physiology (42.5% vs. 17.2%, *p* = 0.013, OR = 16.4), cardiopulmonary exercise testing (52.8% vs. 24.1%; *p* = 0.006, OR = 3.5), and PA benefits (89.6% vs. 34.5%; *p* < 0.001, OR = 3.5). Although most medical students have been taught about PA’s health benefits, only a small proportion have sufficient knowledge for effective prescription.

## 1. Introduction

In recent decades, the prevalence of lifestyle-related non-communicable diseases (NCDs) and the associated mortality rates have been steadily increasing (Naghavi et al., 2017). According to the World Health Organization (WHO), from 2000 to 2019, seven of the ten leading causes of death globally were NCDs, with the top three being ischemic heart disease, stroke, and chronic obstructive pulmonary disease (COPD) (Bull et al., 2020). The economic burden of NCDs is significant, with healthcare costs projected to reach USD 47 trillion worldwide by 2030 (Hacker, 2024).

Among modifiable lifestyle factors, physical activity (PA) has emerged as a cornerstone in the prevention and management of NCDs. Extensive research highlights its benefits, demonstrating that regular exercise lowers blood pressure in normotensive and hypertensive individuals, improves glycemic control in patients with type 2 diabetes, and supports substantial weight reduction in overweight or obese individuals (Edwards et al., 2023; Brittain et al., 2024; Gallardo-Gómez et al., 2024). Additionally, PA is associated with mental health benefits, including reduced depressive symptoms and improved overall well-being (Pearce et al., 2022). Despite robust evidence supporting the multifaceted dose-dependent health benefits of PA (Ekelund et al., 2024; Paluch et al., 2024; Shailendra et al., 2024), physical inactivity remains a challenging global epidemic (Bull et al., 2020; Katzmarzyk et al., 2022).

Healthcare providers (HCPs) play a central role in counseling and educating patients about health and PA (Pearson & Raeke, 2000; Harris, 2023). Research indicates that patients prefer to receive PA advice directly from medical doctors (Thornton et al., 2016), with general practitioners (GPs) being the most trusted source of such information (Schofield et al., 2005). Training future medical doctors in lifestyle medicine early in their careers is essential (Frates et al., 2024). However, medical education has not consistently incorporated practical training on PA prescription. Several studies have highlighted a lack of knowledge and training in PA counseling among medical students globally, particularly in countries such as the United States, United Kingdom, Canada, and Australia (Chew et al., 2019; Radenkovic et al., 2019; Adedokun et al., 2021; Sahlqvist et al., 2022; Sousa et al., 2024). Barriers may include limited curricular space, a lack of faculty with expertise in exercise science, and the underprioritization of preventive health topics (Garry et al., 2002; Butsch et al., 2020). Although international data are growing, there remains limited evidence regarding PA education in the context of Greek medical training. In this context, the aim of the present study is to examine the extent and nature of PA-related education received by medical students across Greece’s seven medical schools. We specifically assess students’ self-reported knowledge, confidence in counseling patients, and awareness of international PA guidelines. Understanding these patterns can inform future curricular development to better prepare medical students to address the growing burden of lifestyle-related chronic disease through evidence-based PA counseling.

## 2. Materials and Methods

### 2.1. Study Population

The study population comprised medical students in their 4th, 5th, and 6th year and those preparing to graduate from Greece’s medical schools. A convenience sampling method was used to recruit participants for this study. National admissions data show that about 900–950 students enter Greek medical schools each year. After accounting for attrition, this yields roughly 2700 eligible students across the three cohorts (4th–6th years) targeted in our study. The sample included 135 students from the country’s seven medical schools. This represents an overall 5% response rate. Of these, 28.1% were studying in Athens (the capital), 20.0% in Thessaloniki (the second-largest city), 11.9% in Patras, 11.1% in Heraklion, 10.4% in Alexandroupoli, 10.4% in Ioannina, and the remaining 8.1% in Larissa.

Participants were informed about their rights, the study’s purpose, and that participation was voluntary with guaranteed anonymity. The principal investigator and the study supervisor were identified in the consent form, with contact details provided for any inquiries. They were also notified of their right to withdraw within two weeks of participation and were offered the option to receive study results after completion. The study received approval from the Scientific Committee of Attikon University Hospital (protocol number ΕΒΔ430/6 July 2022).

### 2.2. Questionnaire

A structured questionnaire, adapted to the Greek context from a similar tool used with a comparable U.S. sample (Adedokun et al., 2021), was used. As the original tool consists of stand-alone items rather than a composite scale, formal psychometric validation was not required. The questionnaire was forward-translated into Greek by a bilingual researcher and reviewed for content relevance and clarity by three faculty members (CT, PK, and AP). Minor linguistic and contextual adjustments were made. The questionnaire was created using the Google Forms platform and was tested for usability and technical functionality by the principal investigator prior to distribution. All questions were presented on a single page. Data collection took place via the Google Forms platform from 11 November 2022 to 20 January 2023. The questionnaire was distributed simultaneously through medical student social media groups, and an identical reminder message was posted in each group 15 days later. Additionally, it was sent to the official email addresses of medical schools to be shared with students via formal communication channels. Follow-up phone calls were made to school administrators, along with reminders as needed after 15 days. Schools promoted the survey via email, newsletters, website announcements, and social media pages.

The first three questions addressed students’ medical school, gender, and year of study. Additional questions assessed whether students had received instruction on the benefits of PA, including the course type (mandatory, elective, or clinical practice), the course name, the year in which this education occurred, and the total hours of instruction. Students were also asked about their confidence in discussing PA with patients, knowledge of recommendations from the World Health Organization (WHO) and the Greek Central Board of Health (KESY), and their perceived importance of further education on PA and health. All questions were mandatory except those related to PA instruction, including course type, the year the course was taught, and the total hours of instruction.

### 2.3. Statistical Analysis

Data were analyzed using IBM SPSS Statistics for Windows, Version 22.0 (Armonk, NY, USA), with both descriptive and inferential statistics conducted. Descriptive statistics summarized the survey responses, while inferential statistics examined relationships between categorical variables using the chi-square test (*χ*^2^). Odds ratios (ORs) with 95% confidence intervals (95%CIs) and effect sizes (phi, *φ*) were calculated to assess the strength of associations between whether students had been taught the value of physical activity (PA) during their studies and their readiness to discuss various PA-related topics with patients. Effect sizes were interpreted as follows: 0.1 indicates a small effect, 0.3 a medium effect, and 0.5 a large effect. Statistical significance was set at *p* ≤ 0.05. The percentages of students who reported having been taught the exercise were adjusted as follows: for analyses of the 5th and 6th years, students in the subsequent years were excluded (n for the 5th year = 86, n for the 6th year = 63). This approach is based on the rationale that a 5th-year student would not have attended courses taught in the 6th year. The missing data in this study were classified as missing completely at random (MCAR). Since they accounted for less than 5% of the total responses, they were handled using the pairwise deletion method (Kang, 2013; Mirzaei et al., 2022).

## 3. Results

The study included 135 medical students, 67.4% of whom were female. Survey results are summarized in Table 1. Most participants were in their 4th (n = 49; 36.3%) or 6th (n = 47; 34.8%) year of study. Most students (n = 106; 78.5%) reported being taught about the value of PA during their medical school courses. Among these, nearly half (n = 53; 50.5%) identified mandatory courses as the context for this instruction, followed by elective courses (n = 29; 27.6%) and clinical practice (n = 11; 10.5%). However, 30.2% (n = 32) had received less than two hours of instruction, with the most common exposure reported in the 2nd (n = 45; 42.5%), 3rd (n = 42; 39.6%), and 4th (n = 44; 42.3%) years.

Among the respondents who reported being taught the value of PA, 48.9% indicated that this occurred in the elective compulsory course “Physiology of Exercise and Ageing”. Additionally, 25% mentioned “Internal Medicine” and 25% identified “Cardiology” as courses where this topic was covered. A smaller proportion, 6.5%, reported the “Nutrition” course, and 5.4% indicated the “Biochemistry” course. Less than 5% of respondents identified other courses, including “Orthopaedics” and “Physical Medicine & Rehabilitation,” as covering the value of PA. The respondents could select multiple courses, as the question allowed for identifying all applicable lessons where the value of PA in health was taught.

Regarding readiness to discuss PA with patients, 77.8% (n = 105) felt confident addressing its benefits, while fewer were prepared to discuss specialized topics like cardiopulmonary exercise testing (CPET; n = 63; 46.7%) and exercise and behavior change (n = 52; 38.5%). Only 26.7% (n = 36) felt ready to prescribe exercise based on individual health conditions, and 14.8% (n = 20) felt unprepared to discuss any aspect of PA. Knowledge of international PA guidelines was limited, with only 25.2% (n = 34) aware of the WHO or KESY recommendations for the general population. While 61.5% (n = 83) were familiar with the recommendation for 150–300 min of moderate-intensity aerobic activity per week, only 40.0% (n = 54) knew about the importance of muscle-strengthening exercises twice a week. One hundred and ten (81.5%) students emphasized the importance of additional education on PA, with 43.0% (n = 58) considering it very important and 38.5% (n = 52) important (Table 1).

**Figure 1:**
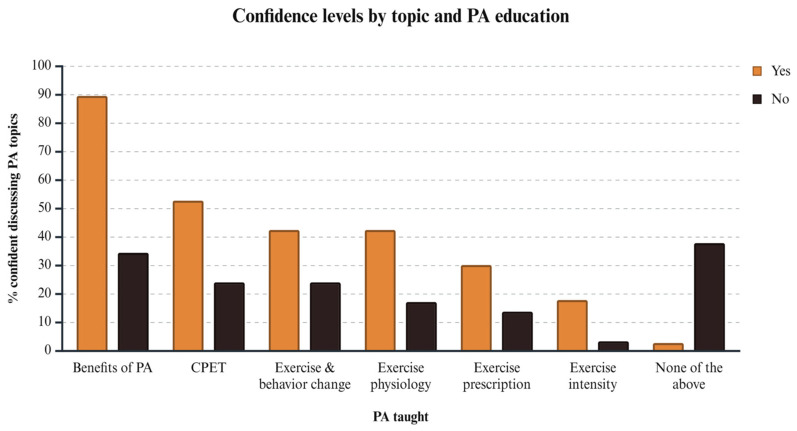
Confidence levels by topic and PA education. Percentage of students reporting confidence in discussing various PA topics with patients, stratified by whether they received formal PA education during medical school. Created in https://BioRender.com.

There was a statistically significant association between the medical school attended and whether students had been taught the value of PA during their courses (*χ*^2^ = 24.441, *df* = 6, *p* < 0.001, *φ* = 0.425), indicating a medium-to-large effect size. Post hoc analysis using the Bonferroni correction revealed a significant difference between the medical schools in Athens and Larissa, with 97.4% of students (n = 36 out of 37) from the Athens medical school reporting that they had been taught the value of PA, compared to only 36.4% of students from the Larissa medical school (n = 4 out of 11). Additionally, a statistically significant association was found between whether the students had been taught the value of PA for health during their studies and the readiness to discuss the benefits of PA (*χ*^2^ = 40.055, *df* = 1, *p* < 0.001, *φ* = 0.545, OR = 16.4 [6.1, 44.1]), CPET (*χ*^2^ = 7.532, *df* = 1, *p* = 0.006, *φ* = 0.236, OR = 3.5 [1.4, 8.9]) and exercise physiology (*χ*^2^ = 6.206, *df* = 1, *p* = 0.013, *φ* = 0.214, OR = 3.5 [1.3, 10.0]) with patients, demonstrating effect sizes ranging from small to large. Specifically, 42.5% (n = 45 out of 106) of students who had received instruction on the value of PA felt prepared to discuss exercise physiology with patients, compared to only 17.2% (n = 5 out of 29) of those who had not received such instruction; the corresponding percentages for readiness to discuss the benefits of PA and CPET were 89.6% vs. 34.5% and 52.8% vs. 24.1% (n = 95 vs. 10 and n = 56 vs. 7, respectively). These associations remained statistically significant after adjusting for gender and year of study (Figure 1, Table 2).

## 4. Discussion

The purpose of this study was to investigate medical students’ perspectives on their education regarding the importance of PA in health. The questionnaire used in this study was adapted from a similar tool developed for U.S. medical schools (Adedokun et al., 2021). The findings revealed significant gaps in both the instruction and knowledge of PA among students. Although almost 80% of respondents reported being taught about the importance of PA during their studies, 30.2% indicated that this occurred in less than two hours of instruction, primarily during the early years of their education, before clinical practice courses began. A small proportion of students felt confident discussing specialized topics with patients, such as behavior change strategies (e.g., motivational interviewing) or exercise prescription parameters. Notably, over 40% of students who had received instruction on PA felt prepared to discuss exercise physiology with patients, compared to only 17.2% of those without such instruction. Additionally, the vast majority of educated students were ready to discuss the benefits of PA (89.6%), compared to almost one-third of uneducated students. Moreover, only 25.2% of students were aware of the current WHO PA recommendations for the general population. Despite these gaps, more than 80% of students emphasized the importance of additional PA education in medical school, recognizing its critical role in health and patient care.

In recent years, an increasing number of studies have characterized regular PA as a therapeutic equivalent to medication. For instance, analyses have shown that mortality rates among individuals with NCD participating in exercise programs do not differ significantly from those undergoing pharmacological treatments (Fiuza-Luces et al., 2013; Naci & Ioannidis, 2015). Moreover, as early as 2004, research demonstrated that participation in exercise programs provided greater benefits for patients with chronic angina compared to invasive coronary artery angioplasty (Hambrecht et al., 2004). Additionally, in some mental health conditions, such as anxiety and depression, PA has been found to offer 1.5 times greater benefits than standard psychotherapy or medication (Dale et al., 2019). This growing evidence has led to the notion of prescribing exercise to prevent or manage individuals with NCDs (Pedersen & Saltin, 2015; Thornton et al., 2016). Consequently, educating medical students about PA is essential, particularly as the primary care setting remains the most trusted source of information on PA for patients (Schofield et al., 2005).

Our study included a sample of 135 medical students from seven medical schools across Greece, representing students in their 4th, 5th, and 6th years and those preparing to graduate. While smaller than the samples in some international studies, our sample size is proportional and comparable given the smaller population of medical students in Greece compared to larger countries. For example, studies conducted in the U.S. included 432 and 480 participants, respectively, reflecting the significantly larger population of medical students in that country (Adedokun et al., 2021; Stoutenberg et al., 2023). Similarly, UK-based studies reported sample sizes of 158 and 633 participants, the latter combining students from both the UK and Singapore (Chew et al., 2019; Radenkovic et al., 2019). In Australia, a country with more than double the population of Greece, a study included 107 participants (Sahlqvist et al., 2022). Despite differences in sample sizes, the findings from our study are comparable to these international studies, highlighting universal challenges in integrating PA education into medical curricula. The inclusion of students from all seven medical schools in Greece strengthens the representativeness of our findings within the context of the country’s medical education system. Additionally, the reporting of effect sizes, which are independent of sample size, adds strength to our analyses by providing context on the practical relevance of observed differences (Fritz et al., 2012).

In our study, over 80% of students considered additional education on PA during their medical training to be at least important, a figure comparable to findings from a UK study where 80% of students expressed a desire for more lifestyle medicine to be incorporated into the curriculum (Radenkovic et al., 2019). Similarly, in a U.S.-based study, 92.4% of students rated formal training in lifestyle medicine approaches as important or very important (Stoutenberg et al., 2023). However, 60.4% of students received less than five hours of PA instruction, with 30.2% receiving less than two hours and another 30.2% receiving between two and five hours. This is higher than a U.S. report, where 35.4% and 47.0% of students received minimal training (no or 1–5 h) in PA (Stoutenberg et al., 2023). Additionally, our findings are consistent with those from other studies indicating that most PA instruction occurs during the preclinical years, before clinical practice begins (Adedokun et al., 2021). While 70% of U.S. respondents reported at least one instance of PA-related instruction (Adedokun et al., 2021), this was slightly lower than the 78.5% reported in our study. Moreover, the significant difference in reported PA education between schools—particularly between Athens and Larissa—may be partially explained by differences in elective course structure and timing. In Athens, the elective Physiology of Exercise and Aging is scheduled for the second semester, increasing the likelihood that students had completed it before survey participation. In contrast, the Larissa course Exercise for the Prevention and Management of Non-Communicable Diseases is offered as a flexible elective during the winter semester of any academic year, allowing more variation in student participation. Post hoc analysis using the Bonferroni correction revealed a statistically significant difference between these two schools, with 97.4% of Athens students reporting that they had been taught the value of PA, compared to only 36.4% of students from Larissa. Additionally, the smaller number of participants from Larissa (n = 11) versus Athens (n = 38) may further limit the representativeness of this comparison and should be interpreted with caution.

The proportion of students in our study who felt confident discussing the general benefits of PA with patients (77.8%) was lower than the 89.6% reported in a U.S. study (Adedokun et al., 2021). This discrepancy may reflect differences in the quality, depth, or consistency of PA education. However, students who received formal instruction on the value of PA were significantly more confident in discussing specific topics, such as exercise physiology, with patients compared to those who did not, a pattern observed in other studies (Chatterjee et al., 2017). Notably, students who were taught the value of PA during medical school were over 16 times more likely to address its general benefits (OR = 16.4), and 3.5 times more likely to discuss CPET and exercise physiology with patients compared to their peers who had not received such education. This large odds ratio reflects a strong association between PA instruction and student confidence; however, it may also indicate the very low baseline confidence among students without formal PA education. While encouraging, such high confidence does not necessarily translate into competence or actual clinical behavior, underscoring the need for further research on the impact of PA training on practice readiness and patient outcomes (Chatterjee et al., 2017). Similarly, previous research has demonstrated that incorporating structured electives focused specifically on exercise prescription significantly improves medical students’ confidence and readiness to integrate lifestyle interventions into clinical practice (Mitchell et al., 2024).

Awareness of international or national guidelines has also been linked to higher confidence in PA counseling (Chatterjee et al., 2017), yet only 25.2% of our participants were aware of current WHO guidelines, indicating a critical gap in their training. Why these findings matter is clear: insufficient PA education during medical training risks leaving future physicians ill-prepared to provide evidence-based lifestyle counseling at a time when NCDs are the leading global cause of mortality. Without adequate skills and confidence in PA prescription, graduates may miss critical opportunities to promote prevention, support disease management, and address patient expectations for guidance on physical activity. This educational gap not only affects patient outcomes but also undermines efforts to integrate preventive strategies into routine care—a priority in modern health systems responding to the NCD epidemic. To address this gap, medical schools should not only increase the quantity of PA education but also enhance its quality and clinical relevance. This includes embedding PA counseling into clinical skill assessments, linking PA topics explicitly to competencies required for graduation, and creating opportunities for students to practice these skills in authentic patient encounters during clerkships. National medical education bodies could also develop clear learning objectives and minimum standards for PA education, ensuring consistency across institutions. Furthermore, partnerships with professional bodies in sports medicine, physiotherapy, or public health could help develop and deliver content, particularly where faculty capacity is limited.

Globally, many medical students report limited opportunities to develop the skills needed for effective PA counseling. In Canada, for instance, only 25% of medical students reported discussing PA counseling with patients (Ng & Irwin, 2013). Furthermore, many students fail to meet the minimum recommended PA levels during medical school, and their PA levels often decline further when transitioning from medical school to residency, potentially compromising physicians’ health and their ability to model and promote PA effectively (Pardo et al., 2016). Additionally, promoting structured wellness programs, including dedicated periods for exercise and nutrition, has been associated with improved academic performance, reduced stress, and greater satisfaction among medical students, highlighting the importance of addressing barriers such as inconsistent schedules and limited opportunities for PA (Deisz et al., 2024). Furthermore, evidence from structured health promotion curricula indicates that healthcare students who participate in comprehensive lifestyle interventions report significant long-term improvements in mood, reduced stress, and overall well-being (Pasarica et al., 2025). Also, a positive association has been observed between PA habits and high academic achievements (Mutlu et al., 2024). Despite this growing global awareness, PA education remains underprioritized in Greek medical curricula. One contributing factor may be the limited presence of faculty with formal expertise in exercise science or lifestyle medicine within medical schools. Additionally, the national medical curriculum is already densely packed with traditional biomedical content, leaving little room for preventive health topics such as PA counseling. These systemic factors likely contribute to the variability and insufficiency of PA-related training across institutions. Incorporating structured curricula in lifestyle medicine topics early in medical training is crucial, as it encourages students’ curiosity and promotes the sustained integration of preventive health strategies into their future clinical practice (Sandefur & Bansal, 2024). Specific strategies could also include embedding PA-related content into existing core clinical rotations (e.g., cardiology, internal medicine, pediatrics), integrating case-based learning and simulation exercises focused on PA prescription and counseling, and offering faculty development programs to equip educators with the necessary skills to teach lifestyle medicine effectively. Such initiatives could better prepare medical students to address the growing burden of NCDs through evidence-based lifestyle interventions. Moreover, medical schools could consider implementing a formal PA education strategy that conducts periodic audits or reviews of PA-related content within the curriculum to identify gaps and ensure alignment with international guidelines (e.g., WHO recommendations).

A strength of this study is the inclusion of medical students from all seven medical schools in Greece, providing a nationwide perspective on PA education in medical curricula. Additionally, the study addresses an important gap in the literature by focusing on a country with limited prior research on this topic. However, there are some limitations to consider. Because the study relied on convenience sampling and achieved a modest 4.5% response rate, selection bias is possible; students already interested in physical activity may have been more likely to participate, which could limit the generalizability of our findings to the wider Greek medical student population. These students may also be more inclined to choose PA-related courses, potentially skewing the results and limiting generalizability to the average medical student. Additionally, the self-reported nature of the survey set a risk of recall bias, particularly for students reflecting on training they received years earlier. This could lead to overestimation or underestimation of the duration and quality of PA instruction. Furthermore, social desirability bias may have influenced responses, as participants could have overestimated their confidence or knowledge of PA topics to align with perceived expectations. The study also lacked methodological triangulation—such as curriculum analysis or qualitative interviews—which could have helped validate the self-reported data and provided a more comprehensive understanding of PA education across institutions. Lastly, the study did not evaluate the practical application of PA knowledge in clinical settings, which is a critical component of medical training that warrants further exploration.

## 5. Conclusions

While most students reported receiving instruction on PA, this was predominantly during their preclinical years, often limited in duration and depth. Although most students recognized the benefits of PA, their ability to provide specific guidance, such as discussing exercise physiology or prescribing exercise based on individual health conditions, was limited. Students who were taught the value of PA during medical school were over 16 times more likely to address the general benefits of PA and 3.5 times more likely to discuss cardiopulmonary exercise testing and exercise physiology compared to their peers who had not received such an education. Awareness of international guidelines, such as those from the WHO, was also low, with only 25.2% of students familiar with these recommendations. The findings suggest that medical curricula should integrate more comprehensive and practical PA-related training, particularly during clinical years, where the relevance to patient care is more apparent. Specific actions could include embedding PA education into core clinical rotations, providing faculty development to enhance teaching capacity in this area, and utilizing simulation or case-based learning to strengthen counseling skills. Future studies could explore the long-term impact of PA education on clinical practice and patient outcomes, particularly as students transition into residency and independent practice. Additionally, intervention-based research evaluating the integration of structured PA training within medical curricula—especially during clinical years—may help identify effective educational strategies to improve PA counseling competence. Cross-national comparisons could also shed light on structural differences and best practices in medical education systems.

## Figures and Tables

**Table 1:** Survey results on physical activity education among medical students (n = 135).

Variable	n (%)
**Sex**	
Male	44 (32.6)
Female	91 (67.4)
**Year of study**	
4th year	49 (36.3)
5th year	23 (17.0)
6th year	47 (34.8)
Preparing to Graduate	16 (11.9)
**Have you been taught the value of PA during medical school courses?**	
No	29 (21.5)
Yes	106 (78.5)
Course type (n = 105) *	
Mandatory	53 (50.5)
Elective	29 (27.6)
Clinical practice	11 (10.5)
Mandatory and Elective	4 (3.8)
All of these	4 (3.8)
Briefly covered in various courses	4 (3.8)
In which year was the course taught? (*selected all that apply*) (n = 104, 2 missing values) *	
1st year	24 (22.6)
2nd year	45 (42.5)
3rd year	42 (39.6)
4th year	44 (42.3)
5th year (n = 86) **	26 (30.2)
6th year (n = 63) **	13 (20.6)
Total hours of instruction (n = 106) *	
<2 h	32 (30.2)
2–5 h	32 (30.2)
5–10 h	22 (20.8)
11–20 h	13 (12.3)
>20 h	7 (6.6)
**Readiness to discuss PA topics with patients (*select all that apply*)**	
Benefits of PA	105 (77.8)
Cardiopulmonary exercise testing	63 (46.7)
Exercise and behavior change (e.g., Motivational interviewing)	52 (38.5)
Exercise physiology	50 (37.0)
Exercise prescription (individualized by health condition)	36 (26.7)
Exercise intensity/Safe and beneficial exercise zones	20 (14.8)
None of the above	14 (10.4)
**Knowledge of current PA guidelines (WHO/KESY)**	34 (25.2)
**Knowledge of specific recommendations for adults:**	
150–300 min of moderate-intensity aerobic PA per week	83 (61.5)
Muscle strengthening exercises ≥2 times per week	54 (40)
**Perceived value of additional education on PA**	
Very important	58 (43)
Important	52 (38.5)
Moderate	21 (15.6)
A little	3 (2.2)
Not at all	1 (0.7)

PA, physical activity; WHO, World Health Organization; KESY, Greek Central Board of Health; * out of the those who were taught the value of PA during the medical school courses (n = 106); ** for analyses of the 5th and 6th years, students in the subsequent years were excluded (n for the 5th year = 86, n for the 6th year = 63).

**Table 2:** Strength of association between readiness to discuss PA topics with patients and whether students were taught the value of PA during medical school courses (n = 135).

	*χ* ^2^	*p*-Value	*φ*	OR [95% CI]	Adjusted OR [95% CI], *p*-Value
Benefits of PA	40.055	<0.001	0.545	16.4 [6.1, 44.1]	17.3 [5.8, 51.7], <0.001
CPET	7.532	0.006	0.236	3.5 [1.4, 8.9]	3.3 [1.3, 8.8], 0.014
Exercise physiology	6.206	0.013	0.214	3.5 [1.3, 10.0]	4.4 [1.5, 13.1], 0.008
Exercise and behavior change	3.562	0.059	0.162	2.3 [0.9, 5.9]	2.5 [0.9, 6.5], 0.072
Exercise prescription	3.130	0.077	0.152	2.7 [0.9, 8.4]	2.5 [0.8, 8.0], 0.127
Exercise intensity	3.781	0.052	0.167	6.1 [0.8, 47.8]	6.8 [0.8, 54.6], 0.072
Nove of the above	30.183	<0.001	−0.473	0.48 [0.12, 0.19]	0.03 [0.006, 0.2], <0.001

φ: phi indicates effect size (0.1 = small, 0.3 = medium, 0.5 = large); OR (95% CI): odds ratio with 95% confidence interval; CPET: cardiopulmonary exercise testing; Adjusted OR: adjusted for gender and year of study.

## Data Availability

The raw data supporting the conclusions of this article will be made available by the authors on request.
